# Risk-sensitivity and the mean-variance trade-off: decision making in sensorimotor control

**DOI:** 10.1098/rspb.2010.2518

**Published:** 2011-01-05

**Authors:** Arne J. Nagengast, Daniel A. Braun, Daniel M. Wolpert

**Affiliations:** 1 Computational and Biological Learning Lab, Department of Engineering, University of Cambridge, Cambridge CB2 1PZ, UK; 2 Department of Experimental Psychology, University of Cambridge, Cambridge CB2 3EB, UK

**Keywords:** motor control, risk, decision-making, mean-variance trade-off, prospect theory

## Abstract

Numerous psychophysical studies suggest that the sensorimotor system chooses actions that optimize the average cost associated with a movement. Recently, however, violations of this hypothesis have been reported in line with economic theories of decision-making that not only consider the mean payoff, but are also sensitive to risk, that is the variability of the payoff. Here, we examine the hypothesis that risk-sensitivity in sensorimotor control arises as a mean-variance trade-off in movement costs. We designed a motor task in which participants could choose between a sure motor action that resulted in a fixed amount of effort and a risky motor action that resulted in a variable amount of effort that could be either lower or higher than the fixed effort. By changing the mean effort of the risky action while experimentally fixing its variance, we determined indifference points at which participants chose equiprobably between the sure, fixed amount of effort option and the risky, variable effort option. Depending on whether participants accepted a variable effort with a mean that was higher, lower or equal to the fixed effort, they could be classified as risk-seeking, risk-averse or risk-neutral. Most subjects were risk-sensitive in our task consistent with a mean-variance trade-off in effort, thereby, underlining the importance of risk-sensitivity in computational models of sensorimotor control.

## 1. Introduction

In the fields of psychology and economic decision-making, it is well established that risk attitudes influence human behaviour. For example, when given a choice between a sure bet of $50 and a 50 : 50 chance of winning $100 or $0, most people would prefer the sure bet, even though on average the two options have the same mean payoff. In fact, a risk-averse decision-maker would even prefer a sure bet with a slightly lower payoff, say $45, and thus accept a $5 risk premium—a fact that is exploited by insurance companies in their policies. By contrast, risk-seeking individuals assign higher value to options that have greater variability—for example, when gambling in a casino. Risk might also play an important role in motor tasks. Consider, for example, a climber who has to choose between different routes—a long secure route or a shorter route that could lead to the goal faster, but could take longer if slippery. On his way he might be faced with many such decisions.

The theory of risk in decision-making goes back to the eighteenth century [1] and has since flourished into a host of different models of decision-making under uncertainty [2–7]. One of the most popular risk models in modern finance is Markowitz' risk-return model, in which the value *U*(*x*) of an investment *x* is modelled as a trade-off between the expected payoff (mean return) 𝔼(*x*) and the variability of the payoff (risk) *Var*(*x*), such that *U*(*x*) = 𝔼(*x*) − *θVar*(*x*). The parameter *θ* expresses the decision-maker's risk attitude: risk-neutral decision-makers are only sensitive to the expected payoff (*θ* = 0), while risk-averse individuals discount payoff variability (*θ* > 0) and risk-seekers consider it a bonus (*θ* < 0). In biology, mean-variance models of risk-sensitivity have been previously applied in ecology [8] and neuroeconomics, elucidating the neural underpinnings of risk-sensitivity in economic choice tasks [9–16]. In psychology and behavioural economics, many other studies have also provided evidence for risk-sensitivity in the context of prospect theory, in which risk is thought to arise through nonlinear distortions of values and probabilities [3].

In contrast, most research on the human motor system has emphasized risk-neutrality and has not considered payoff variance as a potential influence on behaviour. For example, a number of studies have proposed that humans choose movement strategies so as to maximize an average gain in inherently uncertain motor tasks that involve both spatially [17–19] and temporally structured rewards [20,21]. As average gain models only consider mean rewards, they are neutral with respect to risk. Similarly, current computational theories of motor control often consider exclusively mean movement costs and are, therefore, risk-neutral. For example, in most studies on optimal feedback control theory, the optimal behaviour does not consider how variable the movement cost is, but only depends on the average cost [22–27]. Recently, however, violations of the mean payoff hypothesis have been reported in motor control tasks. Wu *et al.* [28] showed, for example, that in a pointing task subjects exhibit risk-seeking behaviour in line with prospect theory, because they systematically underweight small probabilities and overweight large probabilities of hitting designated targets by pointing movements. Similarly, Nagengast *et al.* [29] showed that subjects exhibit risk-averse behaviour in a motor task that required them to control a Brownian particle under different levels of noise. Subjects' changes in control gain depended on their risk-sensitivity in line with the predictions of a risk-sensitive optimal feedback controller [30]. Here, we examine the hypothesis that risk-sensitivity in sensorimotor control tasks can be understood as a trade-off between the mean movement cost and the variability of the cost, analogous to the risk-return model used in economics.

## 2. Methods

### (a) Experimental set-up

Fifteen right-handed subjects (eight male, seven female, aged 20–30) participated in the experiment after providing written informed consent. The experimental protocols were approved by the local ethics committee. Subjects were naive to the purpose of the experiment and none of the subjects reported any sensory or motor deficits. While seated, subjects used their right hand to grasp the handle of a vBOT force-generating robotic manipulandum, which could be moved in the horizontal plane (for details, see [31]). The position and velocity of the hand were computed online at 1000 Hz. Subjects could not see their arm but the position of their hand could be displayed in the plane of the arm using a reflected rear-projection system.

The task was an implicit motor version of a binary economic decision-making task. In the economics domain probabilities and rewards (or losses) are typically both represented explicitly by informing subjects about the numbers involved. In contrast, in our task losses were determined by the effort subjects had to exert to achieve a movement and the probabilities were determined implicitly by the subjects' motor variability. We used a two-alternative forced-choice paradigm in which subjects chose on each trial between a certain fixed effort movement and a gamble in which they would have to make either a lower or higher (than the fixed) effort movement. Which of these efforts they would experience if they chose the gamble was determined probabilistically, with probability *p*_hit_ and 1 − *p*_hit_, respectively. The probability *p*_hit_ was implicitly encoded by the size of a small target region subjects could try to hit in a limited time (with the target size calibrated so that the probability of hitting the target, *p*_hit_, was controlled). If the target was hit, they then made the lower effort movement, but if they missed they made the higher effort movement. Each trial of the experiment, therefore, involved two stages. First, subjects made a choice between a sure and a risky strategy (decision stage) and then produced a movement under the associated effort level (effort stage). The main experimental manipulation was to change the effort levels over trials so as to influence the mean and variance of the effort and study how these changes influence choice behaviour. Subjects were instructed to choose the option that they preferred.

#### (i) Decision stage

**Figure 1.**
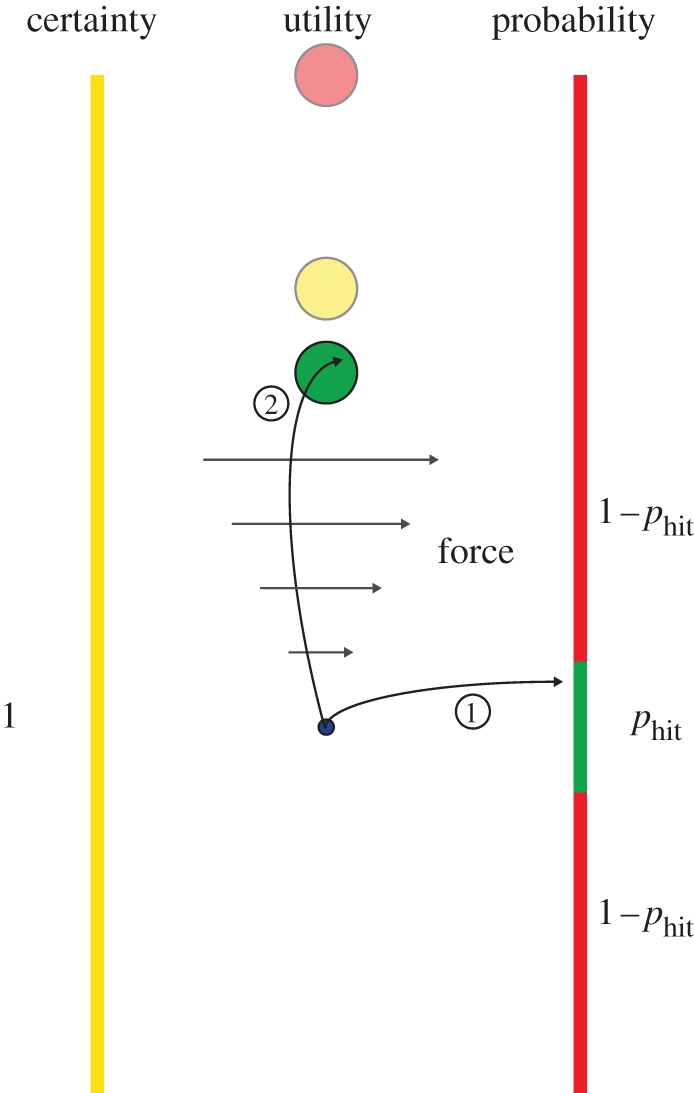
Schematic of experiment. A trial in the ‘mean-variance session’ consisted of two stages: a decision stage and an effort stage. Three possible circular targets were displayed (green, the closest; red, the furthest; yellow, always at 10 cm from the origin). The target selection from these depended on the outcome of the decision stage. (1) In limited time, subjects chose to move their hand (represented by the small blue circle) either to the left or to the right. The left-hand side was a sure bet and the yellow circular target was always selected. Moving to the right was risky and subjects attempted to hit a small green target. Having established the subjects' Gaussian endpoint distribution for this movement previously, a given target size corresponded to a particular probability of hitting the target *p*_hit_. Therefore, if subjects chose the risky strategy they would have a probability of *p*_hit_ of hitting the green target-wall and 1− *p*_hit_ of hitting the red target-wall. The size of the yellow wall was always the same. (2) In the effort stage, subjects moved to the corresponding target where they had to push against a stiff spring requiring a force *F*_right_. We varied the probability *p*_hit_ and the red and green circular target positions to establish for which effort level subjects were indifferent between the sure bet and the risky option for five levels of effort variance.

The decision stage started with three effort circles (green, yellow and red; 0.75 cm radius) being displayed along the vertical axis of the screen (figure 1). The effort circles represented all the possible effort levels that could be experienced by the subject in the effort stage of that trial. The yellow circle was always *x*_yellow_ = 10 cm from the start location (the sure bet), while the test stimuli were represented by the green and red circles, with the green circle always having a shorter distance, *x*_green_ < 10 cm (lower effort), and the red circle always a greater distance, *x*_red_ > 10 cm (higher effort), from the starting location. The colours of the three effort circles corresponded to the colours that were used to indicate different target regions on two walls that were located 20 cm lateral to the starting location and extended the full height of the screen. Subjects moved from the starting location to hit one of the two walls. The left wall was entirely yellow, whereas the right wall was red with a green region embedded whose height was varied between trials (figure 1). The green region determined the probability of *p*_hit_, which was equilibrated in a test session to fit subjects' individual motor variability (compare experimental sessions). Depending on which of the three colour regions subjects hit they would have to move to the corresponding effort circle. Therefore, they could always choose the yellow effort circle if they wished (sure bet) or take the risky option of aiming for the green region and either reach to the green or red effort circle depending on the outcome. To make the task more demanding, the movement time was limited to 0.3 s (if longer, subjects had to repeat the trial) and we introduced a visual gain of 3 in the *y*-direction relative to the starting location (i.e. errors were magnified threefold) and this gain was kept constant throughout the experiment.

#### (ii) Effort stage

After they had made their decision and hit one of the regions, subjects had to move their hand to the corresponding effort circle and hold it there for 1.5 s against a spring-like force *F*_right_ that was pushing them to the right and whose magnitude was proportional to the distance *x* that they were away from the starting location, *F*_right_ = *κ* · *x*. The spring constant *κ* was adjusted to the strength of each subject at the beginning of the experiment. We used body weight as a proxy for maximum force production and the spring constants ranged from *κ* = 125 Nm^−1^ for the lightest (weight approx. 50 kg) to *κ* = 200 Nm^−1^ for the heaviest subject (weight approx. 80 kg). As the spatial range of targets was small we ignore changes in configuration on the arm (biomechanics) affecting the subjective measures of effort.

#### (iii) Experimental sessions

The first 200 trials were a training session, in which subjects practiced hitting the green region on the right-hand side, which varied in size from trial-to-trial (0.5–5 cm, 20 trials each). The next 50 trials familiarized subjects with the different effort levels. Subjects moved to hit the yellow wall anywhere along its length and then moved to the yellow effort circle whose distance varied from trial-to-trial (1–19 cm, five trials each). The subsequent 100 trials (the ‘*σ*-estimation session') was used to estimate subjects' endpoint variability. Subjects attempted to hit a small 0.5 cm green region (equivalent to a range of motion of the hand of 0.5/3 = 0.16 cm owing to the visual gain). The variance of the (approx.) Gaussian endpoint distribution was used to establish the relationship between target size and hitting probability for different target sizes that was used subsequently. The last 400 trials were the test session (the ‘mean-variance session’) in which we measured the subjects' choice behaviour.

#### (iv) Stimulus set for finding indifference points

We wished to examine how variability of the effort affected subjects' choices between the sure bet and the risky strategy. To do this, we wanted to find indifference points where subjects would choose each possibility equiprobably (*p* = 0.5). As we were interested in how variance affects the indifference point we created stimulus sets for the risky choice that had a fixed variance and only varied in the mean—thereby finding the mean for the risky choice to which subjects would be indifferent to choosing the sure bet. To create this stimulus set with a fixed variance that differs only in the mean effort, we manipulated both the hitting probabilities (height of the green region) and effort levels of the risky choice (locations of the red and green effort circles).

We discretized both probability and effort space, computed all possible combinations and selected those combinations that had a particular variance within a given tolerance. The probability of hitting a target *p*_hit_ was discretized into steps of 0.01 (101 levels) corresponding to a set of heights of the green region that depended on the individual subject's variance in pointing. The movement effort was discretized into steps of 0.5 cm with *E*_hit_ ranging from 0 to 9.5 cm and *E*_miss_ from 11.5 to 20 cm, corresponding to the effort for the green and red circles. All possible combinations of *E*_hit_, *E*_miss_ and *p*_hit_ (hence *p*_miss_ = 1 − *p*_hit_) were considered resulting in 20 × 20 × 101 = 40, 400 combinations. The mean effort *μ* = *p*_hit_ · *E*_hit_ + *p*_miss_ · *E*_miss_ and the variance *σ*^2^ = (*E*_hit_ − *μ*)^2^ · *p*_hit_ + (*E*_miss_ − *μ*)^2^ · *p*_miss_ were computed for all combinations. Lotteries with a variance of *σ*^2^ = {1,5,11,17,24} ± 0.5 were selected and saved as five stimulus sets used in the experiment resulting in *n* = {1148,1366,1076,780,713} different stimuli for every set. From these five stimulus sets, we selected those stimuli for presentation during the experiment that would provide maximum information about the subjects' indifference points (mean effort) where subjects would choose equiprobably between the risky strategy and the sure bet strategy. To this end, we selected the stimuli based on a standard adaptive fitting protocol (QUEST) [32,33]. This method selects the next stimulus to lie within the 95% confidence interval of the current estimate of the indifference point based on fitting all the data to a logistic function. The trials for each of the five variance levels were interleaved in a pseudo-random order with a total of 80 trials at each variance level. This procedure produced indifference points for each of the five variance levels.

### (b) Models

To estimate subjects' risk-sensitivity, we modelled decisions made by ideal actor models whose choices were contaminated by noise and we used maximum-likelihood methods to estimate parameters of the ideal actor models. In particular, we considered the mean-variance model and prospect theory to explain subjects' choice behaviour. The noise model for both cases can be found together with the methods for the model comparison in the electronic supplementary material.

#### (i) Mean-variance model

As outlined in §1, the mean-variance model of risk-sensitivity postulates a utility function that contains terms that include both the mean payoff and the variance of the payoff such that *U*_1_(*x*) =−𝔼(*x*) + *θ*_1_ *Var*(*x*), where *x* is the distribution of possible distances to the effort circles and *θ*_1_ is the risk-parameter (risk-averse for *θ*_1_ < 0, risk-neutral for *θ*_1_ = 0 and risk-seeking for *θ*_1_ > 0). Note that the sign of the utility has been reversed since distances are ‘disutilities'. Also note that we can use the distance *x* as a proxy for effort, since the force depends on *x* in a linear fashion and utilities are cardinal up to a linear transform—that is, choices that satisfy the usual rationality axioms can be represented by a utility index that is unique up to a linear transformation. We also use a slightly more general formulation of risk-sensitivity, by including higher order statistics beyond the variance. This can be easily achieved by means of a utility function of the form *U*_2_(*x*) = 2*θ*_2_^−1^ ln𝔼(e^−(1/2)*θ*_2_*x*^) that has the same terms as *U*_1_(*x*) in the first two terms of its Taylor Series expansion (with *θ*_2_ = 4*θ*_1_). Importantly, the same generalization can be used to introduce risk-sensitivity to optimal feedback control models [29,30]. Accordingly, the sure bet in our experiment can be represented as *U*_2_(*x*_yellow_) = −*x*_yellow_ and the risky alternative as *U*_2_({*x*_green_, *x*_red_}) = 2*θ*_2_^−1^ ln(*p*_hit_e^−(1/2)*θ*_2_*x*_green_^ + (1 − *p*_hit_) e^−(1/2)*θ*_2_^^*x*_red_^).

#### (ii) Prospect theory

Unlike the mean-variance approach, prospect theory does not have a single risk-parameter. Instead, prospect theory postulates different value functions *v*^+^(*x*) and *v*^−^(*x*) that distort the objective value of *x* and different probability weighting functions *w*^+^(*p*) and *w*^−^(*p*) that distort the objective probabilities depending on a particular reference point, i.e. depending on whether one deals with gains (+) or losses (−) or both. Risk-sensitivity then depends on the shape of the value function as well as the shape of the weighting function. In our experiment, we exclusively deal with losses, since all outcomes require effort (the reference point is 0 effort). For pure loss prospects, the utility of a prospect with binary outcomes *x*_red_ and *x*_green_ and associated probabilities (1 − *p*_hit_), and *p*_hit_ is given by *U*(*x*) = [1 − *w*^−^(*p*_hit_)] *v*^−^(*x*_green_) + *w*^−^((1 − *p*_hit_))*v*^−^(*x*_red_). To parameterize this decision model, we used a standard value function family proposed by Kahnemann & Tversky [3] *v*^−^(*x*) =−*x*^*α*^ and a common probability weighting function family proposed by Prelec [34] *w*^−^(*p*) = exp[−(−ln *p*)^*γ*^]. The decision model is then determined by the parameters *α* and *γ*. Consequently, we can write the sure bet option as *U*(*x*_yellow_ ) = *v*^−^(*x*_yellow_) and the risky option as *U*({*x*_green_, *x*_red_}) = (1 − *w*^−^(*p*_hit_))*v*^−^(*x*_green_) + *w*^−^(1 − *p*_hit_ )*v*^−^(*x*_red_).

## 3. Results

### (a) Mean-variance indifference points

To test the mean-variance hypothesis of risk for motor control, we designed a probabilistic decision-making task in which subjects could choose between a sure bet—a movement of a fixed effort—or a risky option—a movement entailing either a lower or higher effort (figure 1). By controlling the mean and variance of the effort of the risky option, we found indifference points where subjects chose equiprobably between the sure bet and the risky option (see electronic supplementary material, figure S1 shows the psychometric curves for a typical subject). These indifference points were stable through the course of the experiment—that is they did not shift owing to fatigue, for example—and thus they reflect a stationary choice pattern (see electronic supplementary material, results and figure S2). At the indifference point, the mean effort of the risky choice relative to the fixed effort could be equal (risk-neutral), higher (risk-seeking) or lower (risk-averse). Therefore, risk-averse subjects only accept the risky reach if the mean effort level is lower than the fixed effort alternative, whereas risk-seeking subjects are prepared to take a gamble even at unfavourable odds with the hope for the improbable outcome requiring lower effort than the fixed effort alternative.

**Figure 2.**
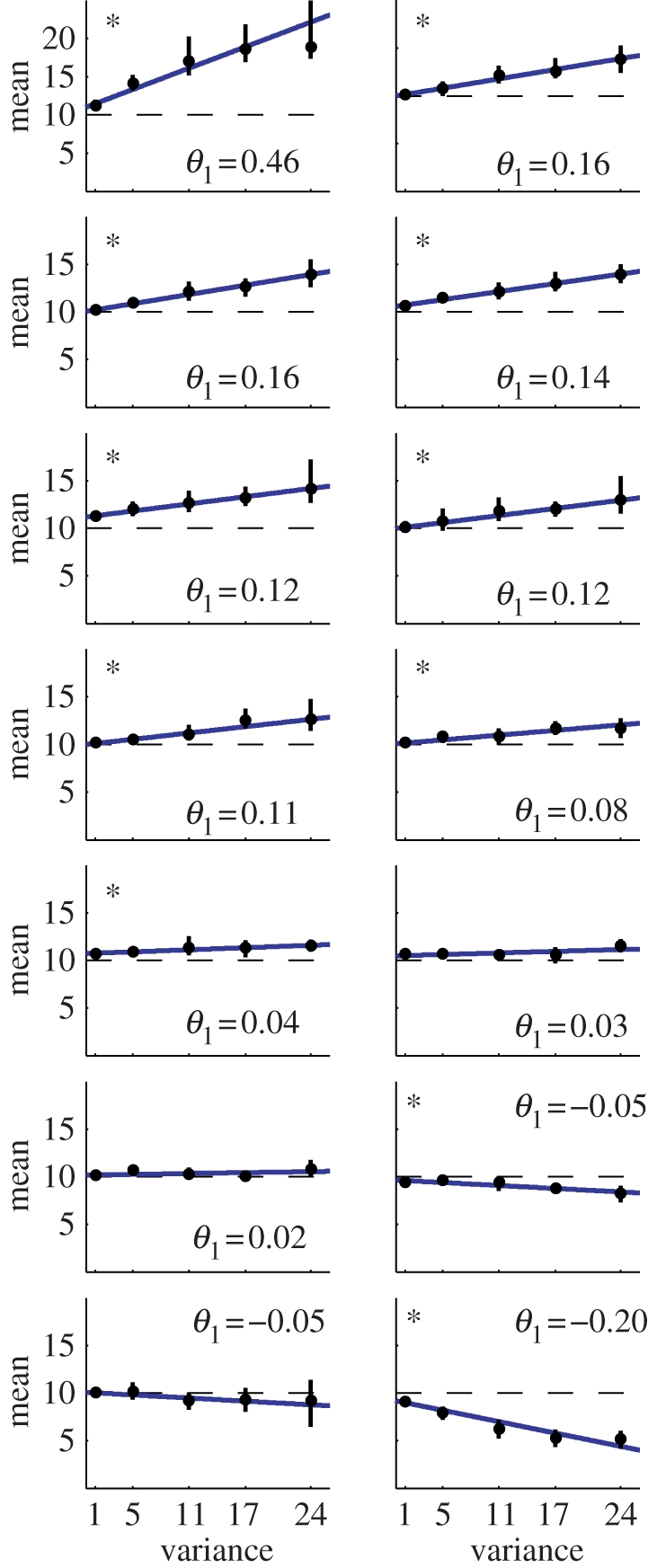
Mean-variance trade-off. The result of the experiment for all 14 subjects ordered from the most risk-seeking to the most risk-averse. The indifference points ±s.d. obtained from the five psychometric curves are shown in black. The best lines of fit obtained using weighted linear regression are shown in blue. The risk-attitude parameter *θ*_1_ is the line's slope and is shown in the right-hand corners of the subplots. For all but three subjects, the null hypothesis of risk-neutrality could be rejected with *p* < 0.05 (marked with an asterisk).

Figure 2 shows the indifference points at the five variance levels for all 14 subjects. We used weighted least-squares regression to obtain linear fits of the five mean-variance indifference points. The slope of these fits informs us about the risk-sensitivity. A slope of zero is compatible with risk-neutrality. A non-zero slope of these fits implies that subjects modulated their indifference points depending on the level of variance. As can be seen by the regressions marked with an asterisks in figure 2, for all except three subjects, the null hypothesis of risk-neutrality, i.e. a line indistinguishable from the horizontal, could be rejected with *p* < 0.05.

### (b) Mean-variance models

The slope of the linear fits allowed us also to infer the risk-parameter in the simple mean-variance model. For the sure-bet reach, the effort circle is always at 10 cm, i.e. *U*_1_^s^ = −E(10) = −10, and for the risky option *U *_1_^r^(*x*) =−𝔼(*x*) + *θ*_1_*Var*(*x*). The curve of indifference points of mean effort levels at different variances can hence be described by the condition *U*_1_^s^ = *U*_1_^r^(*x*) resulting in3.1
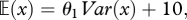
where the slope is the risk-attitude parameter *θ*_1_ we wish to determine, and 10 is expected to be the intercept of the indifference curve. Based on this analysis, we found that the majority of subjects, that is nine subjects, can be classified as risk-seeking in the task, three as indistinguishable from risk-neutral and the remaining two as risk-averse. The risk-attitude parameter *θ*_1_ ranged from 0.46 for the most risk-seeking to −0.2 for the most risk-averse subject (see table 1 for the estimated values). This provides evidence that subjects are not indifferent to the variance of the outcome but have a certain attitude towards risk that influences their decisions.

**Table 1.** Parameter estimates. Mean-variance (*U*_1_). The mean parameter estimates of *θ*_1_ ± s.d. of a mean-variance decision-maker obtained from the linear regression analysis of the subjects' indifference points (see figure 2). Mean-Variance (*U*_2_). The mean parameter estimates of *θ*_2_ ± s.d. (estimated using bootstrapping with 1000 repetitions) of a mean-variance decision-maker obtained using a maximum-likelihood analysis of a noisy decision-maker. Prospect theory. The mean parameter estimates of *α* ± s.d. and *γ* ± s.d. (estimated using bootstrapping with 1000 repetitions) of a prospect theory decision-maker obtained using a maximum-likelihood analysis of a noisy decision-maker.

	subject													
	1	2	3	4	5	6	7	8	9	10	11	12	13	14
mean-variance (*U*_1_)														
*θ*_1_	0.46	0.16	0.16	0.14	0.12	0.12	0.11	0.08	0.04	0.03	0.02	−0.05	−0.05	−0.2
±	0.18	0.03	0.02	0.02	0.03	0.03	0.04	0.03	0.01	0.04	0.05	0.03	0.04	0.06
mean-variance (*U*_2_)														
*θ*_2_	0.43	0.18	0.22	0.25	0.27	0.13	0.16	0.13	0.19	0.1	0.06	−0.18	−0.07	−0.34
±	0.03	0.02	0.02	0.02	0.02	0.02	0.02	0.02	0.03	0.02	0.03	0.02	0.03	0.03
prospect theory														
*α*	0.28	0.12	0.13	0.13	0.22	0.09	0.06	0.09	0.25	0.28	0.12	2.61	2.76	4.76
±	0.05	0.04	0.03	0.04	0.05	0.03	0.03	0.06	0.14	0.18	0.04	0.23	0.45	0.22
* γ*	0.54	1.28	1.45	0.94	0.76	1.67	1.59	2.31	0.88	0.93	2.05	1.22	3.87	1.71
±	0.1	0.14	0.13	0.1	0.08	0.31	0.21	0.26	0.11	0.12	0.17	0.15	0.24	0.08

To check for consistency of the inferred risk-sensitivity parameters, we used a slightly more complex mean-variance model (see §2) to derive risk-sensitivity parameters based on subjects' trial-by-trial choices and then compared the two sets of risk-parameters for all subjects. The ideal actor model assumed a utility function *U*_2_(*x*) = −2*θ*_2_^−1^ln𝔼(e^−(1/2)*θ*_2_^^*x*^), where *θ*_2_ is a risk-parameter. We used a maximum-likelihood method to estimate the parameter *θ*_2_ for each subject (see electronic supplementary material, methods for details). The risk-attitude parameter *θ*_2_ ranged from 0.43 for the most risk-seeking to −0.34 for the most risk-averse subject (see table 1 for the estimated values). The results obtained using the two methods to estimate the risk parameters *θ*_1_ and *θ*_2_ are in good agreement (*ρ* = 0.91, *p* < 0.0001). To test whether this risk-based model was better than a risk-neutral model, we used the Bayesian information criterion (BIC) to compare the ideal actor model to a risk-neutral model. The BIC for the risk-sensitive model was smaller than for the risk-neutral model (risk-neutral decision-maker: BIC = 6256.1, risk-sensitive decision-maker: BIC = 6156.2) supporting the risk-sensitive model and corroborating the findings from the regression analysis of the indifference points. A likelihood ratio test for nested models confirmed the finding of the BIC analysis and showed that the risk-sensitive model fits the data significantly better (*p* < 0.001).

We also fit the risk-sensitive ideal actor model with two different coordinate systems, where forces are not perceived linearly, but nonlinearly either as the square (super-linear) or the square root (sub-linear) of the objective force. The utility model then is given by *U*_2_(*x*) =−2*θ*_2_^−1^ ln𝔼(e^−(1/2)*θ*_2_^^*x*^2^^) and 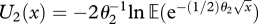, respectively. Importantly, nonlinear transformations of the utility lead to the representation of different preferences. However, the best fits for these nonlinear scales were significantly worse than the best fits with the linear force scale (*p* < 0.001, likelihood ratio test). This suggests that our mean-variance model that assumed an undistorted perception of the experienced forces fits the data better than mean-variance models that assume either super-linear or sub-linear perception of the experienced forces.

### (c) Prospect theory model

**Figure 3.**
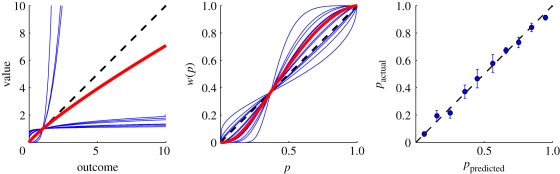
Parameter estimates for the prospect theory fits and control results. (*a*) The estimated value function for each subject (blue) and the mean across subject (red). The dashed line indicates a risk-neutral value function. (*b*) The estimated probability weighting function *w*(*p*) for each subject (blue) and the mean across subject (red). The dashed line indicates no distortion of probabilities. (*c*) The empirical probability of hitting the target in the ‘mean-variance session’ versus the hitting probability predicted by using subjects' endpoint variability from the ‘*σ*-estimation session’ with 1 s.e.m. across subjects. The dashed lines indicates a perfect match between the two.

A different way of looking at human decision-making has been suggested by Kahnemann & Tversky. In their original formulation of prospect theory [3] and its later extension cumulative prospect theory (CPT) [35], deviations from risk-neutrality are due to two factors—the distortion of probabilities in the probability weighting function and the curvature in the value function. In CPT, people's value function is described as convex for monetary losses and concave for monetary gains. In addition, people act as if they misperceive probability, putting too much weight on small probabilities and too little weight on large probabilities. This is captured by a value function and probability weighting function whose shape is determined by a parameter *α* and *γ*, respectively (see §2 for details). We repeated the maximum-likelihood analysis for a CPT decision-maker and estimated the parameters *α* and *γ* (see table 1 and figure 3*a*,*b*). The three subjects that had been classified as risk-averse had convex value functions, the remaining subjects had concave value functions. In general, the estimated *θ*_2_ and *α* were anti-correlated (*ρ* = −0.89, *p* < 0.001). The picture was more mixed for the probability weighting function (*ρ* = −0.43, *p* > 0.05) but the majority of subjects seemed to be under rather than overweight small probabilities (*γ* = 1.51 ± 0.23). Based on BIC, a model comparison with the risk-neutral model was not in favour of the CPT model (risk-neutral decision-maker: BIC = 6256.1, CPT decision-maker: BIC = 6293.9); however, based on the Akaike information criterion (AIC) the CPT model was preferred (risk-neutral decision-maker: AIC = 6163.2, CPT decision-maker: AIC = 6015.4). Comparing the CPT model to the mean-variance model, we found that the mean-variance model was preferred both based on BIC (mean-variance model: BIC = 6156.2, CPT decision-maker: BIC = 6293.9) and based on AIC (mean-variance model: AIC = 5970.6, CPT decision-maker: AIC = 6015.4).

### (d) Control of experimental assumptions

Our experiment depends on the assumption that the subjects' endpoint variability did not change from the ‘*σ*-estimation session’ to the ‘mean-variance session'. This was true for 14 out of 15 subjects (all *p* > 0.3, two-sample Kolmogorov–Smirnov test for the mean-corrected endpoint-distribution of the ‘*σ*-estimation session’ and the ‘mean-variance session'). On average, the endpoint-variability (*σ*) of subjects was 1.90 ± 0.44 cm in the ‘*σ*-estimation session’ and 1.86 ± 0.31 cm in the ‘mean-variance session'. One subject had to be excluded from the analysis as the standard deviation of his movements changed drastically from 5.86 cm in the ‘*σ*-estimation session’ to 1.70 cm in ‘mean-variance session' (*p* < 0.002, two-sample Kolmogorov–Smirnov test). Furthermore, our experimental design relied on predicting the subjects' hitting probability from their endpoint variability. Figure 3*c* shows a plot of the empirical probability of hitting the target in the ‘mean-variance session’ versus the hitting probability predicted by using subjects' endpoint variability from the ‘*σ*-estimation session'. Using linear regression on the data after subtracting the diagonal and testing for zero slope (*t*_8_ = 1.08, *p* > 0.3) and zero intercept (*t*_8_ = 0.9, *p* > 0.3) suggests coincidence of the data with the diagonal and hence confirms accurate prediction of hitting probabilities during the experiment.

## 4. Discussion

In our study, we examined whether subjects are sensitive to the variance of movement costs rather than just the mean level of movement costs. In particular, we investigated how subjects trade off the mean effort against the variability of effort during a movement. Compared with the baseline of a fixed certain effort, we found that most subjects were prepared to accept a gamble with higher mean effort when variability was high (risk-seeking), whereas some were risk-neutral and a minority would only accept a lower mean effort (risk-averse). Our results are consistent with a risk-sensitive decision-maker that trades off the mean and variance of movement effort, but inconsistent with a risk-neutral account of motor control.

A number of previous studies have found that people maximize expected gain in movement tasks in which subjects made speeded pointing movements and the spatial [18,19] or temporal outcome [20,21] of their movement resulted in a monetary payoff. These studies compared subjects' behaviour with an ideal actor model that maximized expected payoff. Crucially, the optimal movement strategy suggested by such models is independent of the variance of the payoff. This should, however, not be confused with the variance of the movement outcome (see electronic supplementary material, discussion for mathematical details). The fact that various kinds of movement variability play an important role in the choice of suitable movement strategies is well known [17] and taken into account by expected gain models. This raises the question as to why these previous studies have not reported risk-sensitivity. One key difference from our study is that in these previous studies the mean and variance of the reward were not manipulated independently of each other making it difficult to establish the effect of one variable alone on subjects' behaviour. Implicit in the ‘gain-maximization hypothesis’ is also that the utility of money is linear across the whole range and not concave for gains and convex for losses as is the usual consensus in behavioural economics [3]. A possible reason why the linear utility function is successful is that these studies used very small monetary remunerations of only a few cents (2.5 cents maximum reward per trial and 12.5 cents maximum loss per trial [18,19,36]). That is they effectively only tested subjects over a very narrow (possibly linear) range of their utility functions. Indeed, a recent study that used larger rewards reported the same value function for money in movement tasks as in economic decision-making tasks [28] and is at odds with the ‘expected gain maximization' hypothesis.

Wu *et al.* [28] examined violations of expected utility theory in a motor task that involved making accurate pointing movements. In particular, they investigated violations of the so-called independence axiom, stating that preferences should not be affected by the addition of a ‘common consequence’. Consider two different tasks in which subjects can choose between lotteries of the form [*p*_1_*U*($*V*_1_), *p*_2_*U*($*V*_2_), …] where there is a probability *p*_1_ of receiving $*V*_1_ that has a subjective utility of *U*($*V*_1_), etc (we assume without loss of generality that *U*($0) = 0). In the first task, we can choose between two lotteries [0.33*U*($2500), 0.67*U*($0)] and [0.34*U*($2400), 0.66*U*($0)]. In a second task, we can choose between [0.33*U*($2500), 0.66*U*($2400), 0.01*U*($0)] and [0.66*U*($2400), 0.34*U*($2400)] = [1.0*U*($2400)]. These two tasks only differ in their ‘common consequence’ in that the second task simply adds 0.66*U*($2400) to both lotteries in task 1. However, in the first task, people tend to prefer the first lottery implying that 0.33*U*($2500) > 0.34*U*($2400) whereas in the second task they tend to prefer the second lottery as it has a guaranteed outcome. Therefore, some decision-makers reverse their preference between the tasks. Importantly, expected utility theory does not allow preference reversals of this kind. Wu *et al.* [28] observed, however, exactly this kind of preference reversals violating the independence axiom. By introducing common consequences in their task, Wu *et al.* [28] simultaneously changed the mean and the variance of their payoffs. In contrast, in our experimental design we did not use common consequences and instead were able to fix the payoff variance of the risky lottery and only change its mean payoff. By examining subjects choice between this risky lottery and the certain lottery (zero variance and fixed payoff), we could directly measure indifference points (for five different levels of variance) where subjects chose equiprobably between the two lotteries. This separate manipulation of mean and variance allowed us to directly show that subjects trade off mean and variance of movement costs.

To compare our results to Wu *et al.* [28], we also fit a prospect theory model to our data, where risk-sensitivity depends both on the distortion of the probability weighting function and the curvature of the value function. Similar to their results, our fit indicated that small probabilities were underweighted in most subjects and that the value function was mostly concave, both of which is consistent with risk-seeking behaviour. However, whether the brain represents risk in agreement with either the mean-variance approach or with the prospect theory account is currently subject of an ongoing debate [37]. Recent evidence from electrophysiological and functional imaging studies has provided support for both theories. In support of the mean-variance approach, separate encoding of reward magnitude and risk has been reported in humans [14–16] as well as in non-human primates [38]. However, recent studies have also found neural evidence in favour of prospect theory. Martino *et al.* [39], for example, reported neural correlates of the framing effect, that is the susceptibility of the decision-maker to the manner in which options are presented. In addition, Hsu *et al.* [40] found that neural responses in the brain depended on probabilities in a nonlinear fashion during a risky task. Both effects are cornerstones of prospect theory. In our experiment, the model comparison favours the mean-variance approach. However, further studies are needed to elucidate how the brain represents value and how the brain′s different valuation and action selection system interact and vie for control to arrive at an overt behavioural decision [41].

Current computational accounts of motor control-like optimal feedback control theory are risk-neutral [26,27] and only consider minimization of the expectation of a cost function, usually with terms for positional accuracy and effort. The variance of the cost does not influence these models when computing the optimal movement policy. However, models of risk-sensitive optimal feedback controllers are compatible with a mean-variance trade-off in movement costs as found in the current study, because the first two terms of the Taylor expansion of the risk-sensitive cost function correspond to mean and variance of the movement cost. Recently, we have shown how risk-sensitive optimal feedback control can account for sensorimotor behaviour under uncertainty in a continuous motor task where subjects had to control a Brownian particle under different noise levels [29]. In this previous study, we found that subjects showed mostly risk-averse behaviour, whereas in the current study and in the study by Wu *et al.* [28] subjects were mostly risk-seeking. An important difference between these experiments is that in the previous study the noise was given by the Brownian particle, whereas in the current study (and also in [28]) the noise was given by subjects' own motor noise. In non-motor settings, the ‘illusion of control' [42] is one of the core factors in causing people to mistake games of pure chance with games of skill even though they are not controllable [43]. Hence, a possible explanation for the difference in risk-sensitivity in our case might be that subjects are risk-seeking because they tend to be over-confident about their own generated motor noise, but risk-averse with respect to noise that is given in their environment. This hypothesis could be tested in future experiments.
